# Internet addiction, loneliness, and academic burnout among Chinese college students: a mediation model

**DOI:** 10.3389/fpsyt.2023.1176596

**Published:** 2023-08-17

**Authors:** Junwang Gu, Ping Wu, Yating Luo, Xiongkun He, Lei Fu, Hanjie Liu, Fen Lin, Qi Xu, Xuanhui Wu

**Affiliations:** School of Public Health and Health Management, Gannan Medical University, Ganzhou, Jiangxi Province, China

**Keywords:** academic burnout, Internet addiction, loneliness, mediation model, structural equation modeling

## Abstract

**Background:**

The dynamics of education and student life have changed since the COVID-19 pandemic. Our society, especially the education system, has become largely dependent on the Internet. This paradigm shifts largely took place in the last few decades. As such, there are various ways in which we cannot comprehend the impact that the Internet can have on student psychology, and how multiple other factors could influence that. Internet addiction and its relationship with academic burnout, along with the impact of loneliness, are all essential factors that must be discussed candidly in the post-COVID-19 era. Hence, the objective of this study was, therefore, to explore the relationship between Internet addiction, loneliness, and academic burnout among Chinese college students as well as the mediating role of loneliness.

**Methods:**

We conducted a cross-sectional questionnaire survey at a Chinese university from October to November 2022. In total, 810 valid respondents were selected via random cluster sampling using the well-established Internet Addiction, Loneliness, and Academic Burnout Scale. The primary approach of mediation analysis and structural equation modeling testing examined the relationships among the three components.

**Results:**

Internet addiction could be responsible for academic burnout among college students. Loneliness partially mediates the relationship between Internet addiction and academic burnout. In a mediated way, different types of loneliness contribute to different types of academic burnout.

**Conclusion:**

Psychological interventions for loneliness, especially emotional loneliness prevention, are the critical aspects of the problem of Internet addiction accompanied with academic burnout. The causal relationship between Internet addiction and academic burnout, possibly of a two-way nature, needs to be further explored in the next future.

## 1. Introduction

The usage of Internet-associated computers, smartphones, and other electronic devices has dramatically increased in the era of social distancing. The expansion of the Internet has led to Internet addiction, usually described as contentious, compulsive Internet use (1). Frequent usage of Internet applications can help minimize negative emotions (2). Internet addiction may damage physical health and lead to a variety of personality and behavior abnormalities in various life domains over a prolonged period (1). According to the 11th revision of the International Classification of Diseases (ICD-11) by the World Health Organization (accessed in 2022), two disorders, owing to online addictive behaviors, were diagnosed: gaming and gambling disorders. Problematic social networks use and problematic shopping behavior can be diagnosed as “other specified disorders due to addictive behaviors (3). Increasing digital addiction has been observed in the past two decades and has dramatically worsened during the COVID-19 pandemic (4). According to published literature (5), global pooled prevalence estimates were 26.99% for smartphone addiction, 17.42% for social media addiction, 14.22% for Internet addiction, and 6.04% for game addiction.

Although the World Health Organization (WHO) announced on May 5 that COVID-19 was now an established and ongoing health issue that no longer constitutes a public health emergency of international concern (PHEIC), the pandemic has profoundly changed the dynamics of education and student life of China over last 3 years. Chinese college students have become more dependent on online learning and entertainment. Reportedly, prolonged exposure to screens and Internet appliances could increase stress and anxiety associated with quarantine time and lockdown, eventually leading to exhaustion and burnout (6). A symptom associated with ill mental health is burnout syndrome (7). An online survey in China showed that 28.4% of participants developed Internet addiction, which correlated with academic burnout (8). Nevertheless, some studies observed that the correlation between excessive use of the Internet and burnout did not exist (9). This possible association between Internet addiction and burnout merits further investigation.

Many studies have tried to ascertain Internet addiction behavior’s psychological correlates (10–12). Psychological mechanisms lead to excessive Internet dependence. The *Theory of Reinforcement* (13) holds that one feels spiritual satisfaction and pleasure when surfing the Internet – going online, distracting from reality, and forgetting the troubles in life; all of which eventually strengthens surfing behavior (14, 15). *Inadequate-personality theory* posits that one may produce personality trait like loneliness when facing real-life problems, and ultimately become vulnerable to excessive Internet use (16, 17). Besides, other models from the diverse perspectives of *Social Learning Theory*, *Cognitive Behavioral Theory*, culture, genetics, and neurobiology have been used to characterize behavioral addiction to the Internet. Internet addiction can be reinforced by psychological factors and simultaneously lead to the development of loneliness (18).

Loneliness is a discrepancy between desired and real social relations (19). The concept of loneliness is multidimensional and complex. It comprises two dimensions: emotional and social (19–21). In general, emotional loneliness is characterized by the absence of an attachment figure (as well as feelings of isolation), and social loneliness refers to the lack of a social network that allows one to develop a sense of belonging, company, and being part of a community (20). The feeling of loneliness is more common during one’s youth. Addiction to the Internet is often linked with a proliferation of loneliness. Meta-evidence demonstrates a moderate positive association between Internet addiction and loneliness worldwide (22). Some scholars believe that the link between Internet addiction and loneliness may be bidirectional, and the causal direction of them might be partly due to the influence of the established model and the chosen method (23). Other researchers, however, have argued that extensive use of the Internet isolates users from the reality of the world (24). Such users conceive an unctuous and weak network of relationships at the cost of real-world ties; consequently, loneliness is a by-product of heavy Internet use (24).

The term “*Academic Burnout*” or “*Student Burnout*” can be traced back to Maslach’s work on “job burnout” (25). Academic burnout refers to students having a negative attitude toward their schoolwork and exhibiting the following behaviors: a decrease in enthusiasm for schoolwork and school activities, as well as indifference and alienation toward classmates and friends owing to long-term pressure from academic curriculums and other aspects (26, 27). The correlation between loneliness and burnout has been reported in some professions. Research showed that loneliness could be employed to predict occupational burnout in medical personnel (28). The national survey of practicing family medicine physicians demonstrates that loneliness is common and is significantly associated with burnout and depression (29). Similarly, a cross-sectional study revealed that loneliness contributed critically to burnout among Canadian workers during the third wave of the COVID-19 crisis (30). However, the effect of students’ loneliness on academic burnout is yet to be reported.

**Figure 1:**
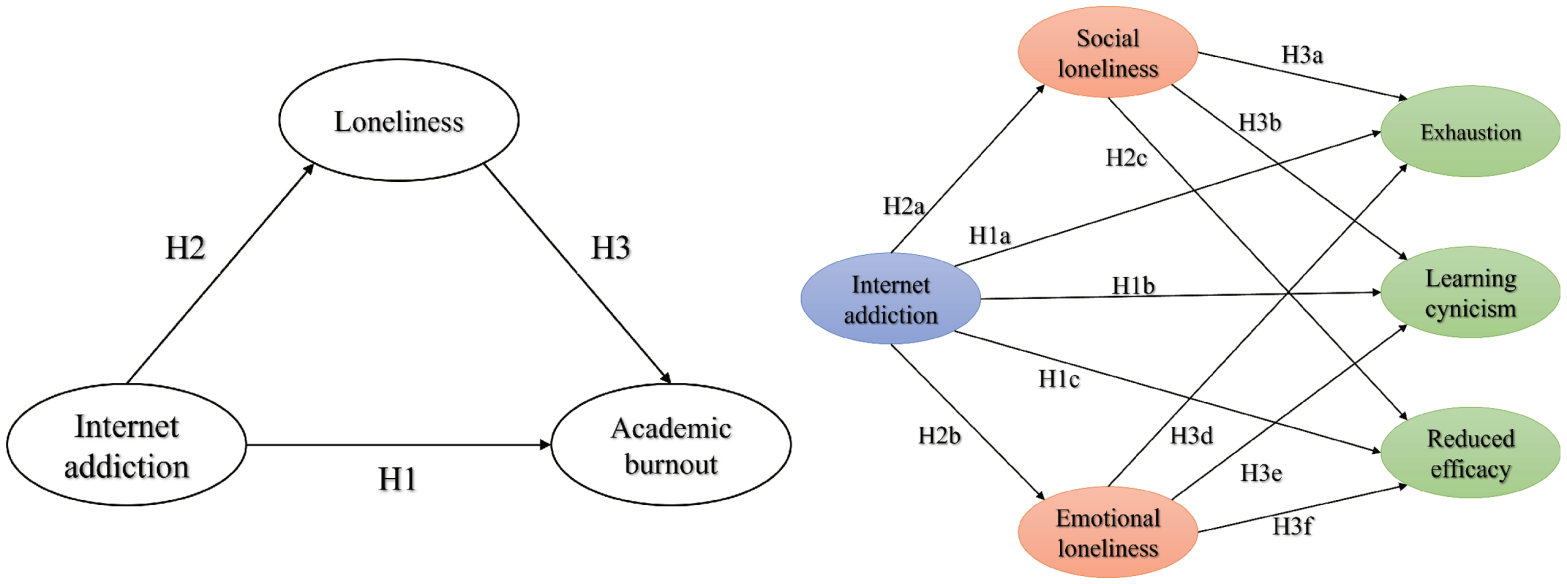
Theoretical hypothesis model linking Internet addiction to loneliness and academic burnout.

Research shows that Internet addiction, loneliness, and academic burnout are common among Chinese college students (31–33), and for college students in China, the prolonged lockdown and the demands of online learning have led to an increased dependence on the Internet (34, 35). While previous studies have revealed the positive relationship between Internet addiction, loneliness, and academic burnout, possible mediating variables influencing this relationship have largely been ignored. Internet addiction, loneliness, and academic burnout may be mutually causal, and exploring their relationship is crucial to designing interventions to minimize students’ addictive behaviors and mental indisposition. However, to date, studies concerning the interaction between them have been limited. Thus, the three theoretical hypotheses derived from the literature mentioned above are: (1) internet addiction could positively cause academic burnout; (2) internet addiction could positively contribute to loneliness; (3) loneliness could positively promote academic burnout. Based on these, we establish the mediation theoretical model (Figure 1) of the relationship between the three factors (Internet addiction, loneliness, and academic burnout) and perform an empirical test, enabling efficient preventive measures for Internet addiction risks, psychological issues, and academic burnout among college students.

## 2. Methods

### 2.1. Respondents

We performed random cluster sampling to select respondents from October to November 2022 at a Chinese University. The specific method is used to randomly select students’ dormitory number and administer questionnaire survey on four students in each dormitory. Participants who were physically healthy and interested in this study were recruited and filled out the questionnaire. All students who were present on the day of the study were included. Those respondents who did not cooperate would be excluded. In total, 836 questionnaires were distributed. Of these, 26 students were excluded as incomplete or erroneously completed questionnaires. Finally, 810 valid responses were obtained with an efficiency recovery rate of 96.89%. The questionnaire consisted of two parts: (1) basic information of the respondents who participated in the survey and (2) instruments of Internet addiction, Loneliness, and Academic burnout.

The study protocol was approved by the ethics review board of Gannan Medical University and was conducted in accordance with the ethical guidelines of the Declaration of Helsinki and relevant policies in China. Informed consent forms were obtained from the participants, which stated that the questionnaire was completed anonymously and the information provided was confidential.

### 2.2. Instruments

***Internet addiction***: the CIAS (36, 37) is a self-report questionnaire developed from the *Chen Internet Addiction Scale* (CIAS) and designed to assess Internet addiction symptoms and associated problems in the Chinese population. The 26-item (A1–A26) questionnaire measures Internet addiction on five dimensions: tolerance, compulsive use, withdrawal symptoms, interpersonal and health-related problems, and time management problems. A four-point Likert scale was used for each item, with 1 representing “no match at all” and 4 representing “definitely match” regarding respondents’ experience in the past 3 months. CIAS-R scores range from 26 to 104, with 64 recommended as the cut-off score (36); a score of 64 or higher on the CIAS-R indicates Internet addiction. According to Cronbach’s alpha, both the total scale (alpha = 0.949) and the subscales of CIAS showed good internal consistency.

***Loneliness***: Russell’s *University of California at Los Angeles Loneliness Scale Version 3* (UCLA-3) was employed to measure participants’ subjective perception of loneliness (38). Participants responded to 20 items (B1–B20) on a 4-point Likert ranging from 1 (*Never*) to 4 (*always*). The higher the score, the higher the loneliness; values >=45 represented high loneliness, 39–44 moderately high loneliness, 34–38 moderate loneliness, 28–33 moderately low loneliness, and 0–27 low loneliness. The scale includes two dimensions: social loneliness and emotional loneliness (39). In this study, the scale had good reliability (Cronbach’s alpha = 0.895).

***Academic burnout***: a self-assessment scale based on the Maslach Burnout Questionnaire was compiled by Chinese scholars of Wu Yan and Dai Xiao-Yang (40). The *Adolescent Student Burnout Inventory* (ASBI) was adopted to measure academic burnout in Chinese adolescent students. A five-point Likert scale ranging from “1 = *totally disagree*” to “5 = *totally agree*” comprises three dimensions: exhaustion, learning cynicism, and reduced efficacy, with 16 items (C1–C16). Cronbach’s alpha was 0.851 based on the present sample of Chinese college students.

### 2.3. Statistics

IBM SPSS (version 20.0, United States) was employed for descriptive statistics and correlation analysis to summarize the principal results. The mediation effect among *loneliness*, *Internet addiction, and Academic burnout* was explored by operating Model 4 of the SPSS Macro PROCESSv3.3 Plug-in compiled by Hayes (41, 42). Confirmatory factor analysis (CFA) and structural equation model (SEM) analyses were conducted using SPSS AMOS (version 26.0, IBM Analytics, United States) software. The bootstrap method was performed to estimate the 95% confidence interval with the number of bootstrap samples of 5,000. Statistical significance was at *p* < 0.05. Pearson’s correlation coefficients (*r*) were calculated with values of 0.1–0.3 considered of small significance, 0.3–0.5 moderate, and 0.5–1.0 large (43).

The sample size of 810 was considered adequate for the SEM analyses; the minimum acceptable sample size recommended for conducting an SEM approach is 200 samples (44). For questionnaire validity and reliability, CFA testing, the index of composite reliability (CR) and average variance extracted (AVE), and Cronbach’s alpha were employed. The chi-square/degree of freedom ratio (*χ*^2^/df), root mean square residual (RMR), incremental fit index (IFI), the Tucker-Lewis Index (TLI), confirmatory factor index (CFI), root mean squared error of approximation (RMSEA) were adopted to determine whether the models fit the data. A rule of thumb for the *χ*^2^/df is that values equal or less than 5 are acceptable fit (45). An acceptable value for CFI, IFI, and TLI is 0.90 and above; while for RMR and RMSEA, it must be below 0.08 (45).

## 3. Results

### 3.1. Respondent characteristics

The characteristics of the respondents are presented in Table 1. A total of 810 respondents (393 men, 417 women) aged 18–24 years (The mean and standard deviation were 18.99 and 1.11, respectively) from a medical university in mainland China participated in this survey. Of the 810 participants, 456 were freshmen (56.4%), 233 sophomores (28.8%), 83 juniors (10.3%), and 36 seniors (4.5%). Two students missed their grade information. Approximately 39.7% of the respondents lived in urban areas, while the rest (60.3%) lived in rural towns. Of the participants, 14.5% were the only child; 1.1% had a monthly allowance of <500 Yuan, 18.9% of 500–1,000 Yuan, 53.0% of 1,000–1,500 Yuan, and 27.0% of ≥1,500 Yuan. 93.3% of students were majoring in science. According to the relevant scale classification, 19.9% of respondents were considered to be addicted to the Internet, and 32.8% were experiencing high loneliness.

**Table 1:** Characteristics of respondents.

Variables	Category	Frequency	Valid percentage (%)
Gender	Men	393	48.5
	Women	417	51.5
Grade of a college	First year (freshmen)	456	56.4
	Second year (sophomores)	233	28.8
	Third year (juniors)	83	10.3
	Fourth year (seniors)	36	4.5
Where is your home?	Urban	321	39.7
	Rural town	487	60.3
Only child?	Yes	117	14.5
	No	690	85.5
Monthly allowance (Yuan)	<500	9	1.1
	≥500 and <1,000	153	18.9
	≥1,000 and <1,500	429	53.0
	≥1,500	218	27.0
Subject category	Students of liberal arts	54	6.7
	Students of science	750	93.3
Internet addiction (score)	Yes (≥64)	161	19.9
	No (<64)	649	80.1
Loneliness (score)	Low loneliness (0–27)	77	9.5
	Moderately low loneliness (28–33)	124	15.3
	Moderate loneliness (34–38)	168	20.7
	Moderately high loneliness (39–44)	175	21.6
	High loneliness (>=45)	266	32.8

### 3.2. Correlations of loneliness, internet addiction, and academic burnout

Prior to all examinations, preliminary assumption testing was employed. A variable is regarded as obedience to normal distribution if univariate skewness is smaller than ±2 and kurtosis is smaller than ±3 (46). As shown in Table 2, the variables of loneliness, Internet addiction, and academic burnout are all in the normal distribution. Internet addiction was positively associated with loneliness (*r* = 0.382, *p* < 0.001) and academic burnout (*r* = 0.534, *p* < 0.001), and loneliness (*r* = 0.578, *p* < 0.001) were positively associated with academic burnout.

**Table 2:** Descriptive statistics and the correlations between variables.

Variables	Internet addiction	Loneliness	Academic burnout
Internet addiction	–		
Loneliness	0.38***	–	
Academic burnout	0.53***	0.58***	–
Range	26–104	20–80	16–80
Mean	53.39	39.97	40.22
SD	13.01	9.22	9.06
Skewness	−0.13	0.15	−0.14
Kurtosis	0.09	−0.08	0.08

***p < 0.001; SD, standard deviation.

### 3.3. Mediation analysis

**Figure 2:**
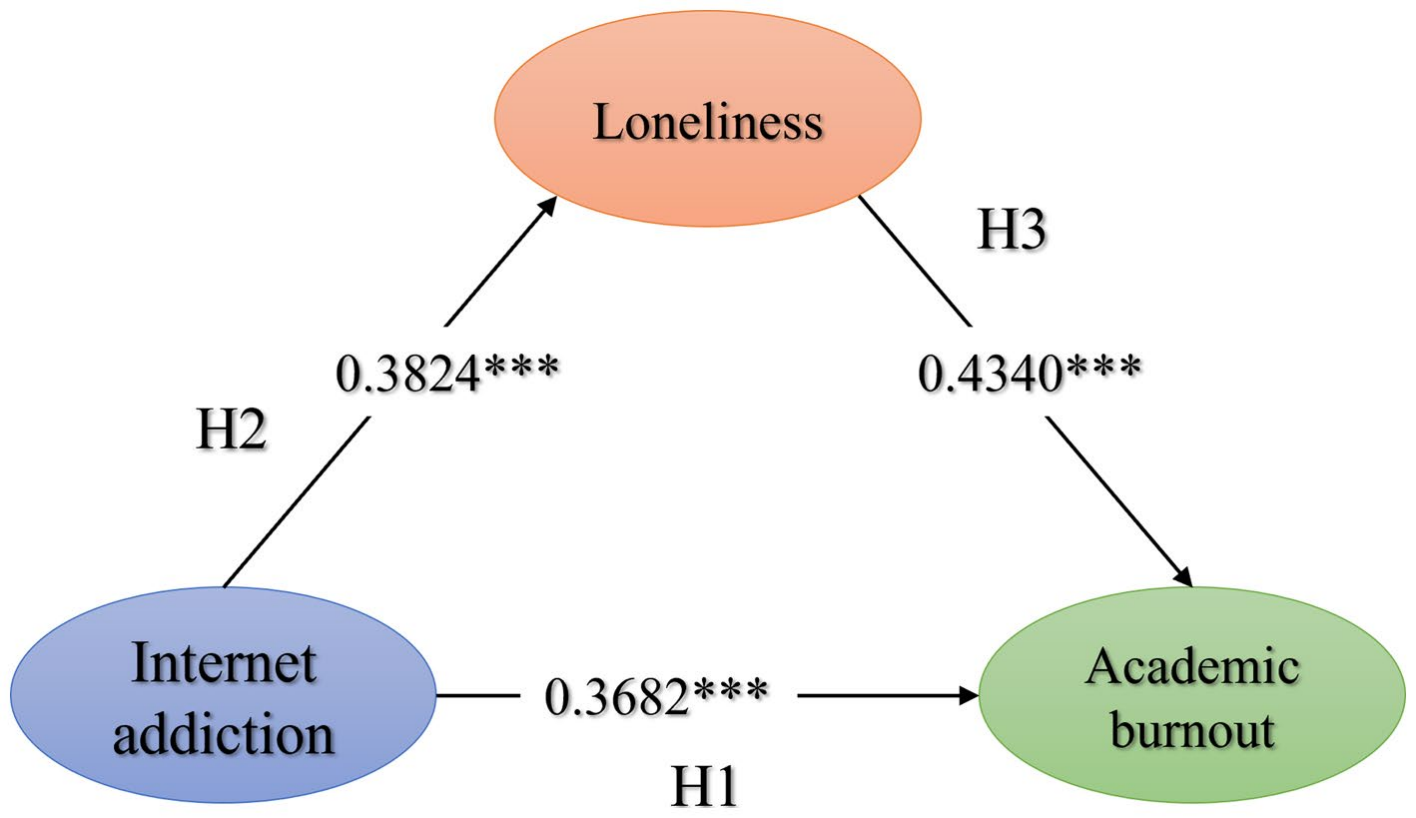
Mediation models. Path coefficients are presented in standardized units, *n* = 810; ***p < 0.001.

As shown in the mediation models of Figure 2, Internet addiction could positively promote academic burnout (*β* = 0.5341, *p* < 0.001). Besides direct effects (*β* = 0.3682, *p* < 0.001), Internet addiction could also indirectly lead to academic burnout through the mediating role of loneliness (*β* = 0.1660, bootstrap 95% CI [0.1272, 0.2080]). The mediating effect contributed to 31.07%, suggesting that loneliness has a partial mediating effect between internet addiction and academic burnout.

### 3.4. CFA testing

The preceding mediated analysis by Process v3.3 suggests loneliness plays a key role in Internet addiction, which leads to learning burnout. However, the model did not analyze a structural equation model (SEM) containing latent variables for each dimension and was unable to examine measurement variable errors; therefore, it was less accurate than a SEM with latent variables. Furthermore, the initial mediation model could not provide model-fitting parameters for overall model evaluation (47). Moreover, the construction of structural equation models with different dimensional variables helps further explore this mediation model’s internal mechanisms. For aforementioned reasons, we further constructed the SEM.

First, we tested the affiliation between the measurement and latent variables using Confirmatory Factor Analysis (CFA). Some items (B16, B17, C7, C5) were removed because of low loading values. Specifically, the items with standardized factor loadings of <0.5 were deleted step-by-step.

The five-dimensional variables of Internet addiction were highly correlated (inter-dimensional variables correlation coefficient all >0.07), suggesting that it is not appropriate to construct models with each individual dimension. Thus, Internet addiction was seen as an overall latent variable. Considering that the Internet addiction scale contains 20 questions, we packaged all measured variables for each dimension into one for easy analysis. Ultimately, the model contains 5 latent variables, namely Internet addiction, social loneliness, emotional loneliness, exhaustion, learning cynicism, and reduced efficacy, with a total of 37 items (Table 3).

**Table 3:** Assessment of normality and reliability.

Variable	Latent variable	Standardized loading factor	Assessment of normality				Cronbach’s Alpha
			Skew	c.r.	Kurtosis	c.r.	
TL	Internet addiction	0.799	−0.054	−0.630	0.113	0.658	0.927
WS		0.823	0.068	0.793	0.315	1.832	
CU		0.915	−0.043	−0.495	−0.216	−1.252	
IHP		0.877	0.030	0.343	−0.137	−0.796	
TMP		0.824	0.222	2.580	−0.099	−0.574	
B20	Social loneliness	0.756	0.737	8.565	0.014	0.082	0.864
B19		0.773	0.714	8.298	0.167	0.967	
B15		0.603	0.523	6.077	−0.429	−2.494	
B10		0.751	0.709	8.236	0.243	1.410	
B9		0.540	0.507	5.889	−0.175	−1.017	
B6		0.663	0.555	6.453	0.161	0.934	
B5		0.733	1.293	15.020	1.387	8.058	
B1		0.531	1.672	19.429	2.330	13.533	
B18	Emotional loneliness	0.556	0.237	2.755	−0.598	−3.474	0.897
B14		0.713	0.015	0.169	−0.588	−3.417	
B13		0.736	0.142	1.652	−0.772	−4.487	
B12		0.605	0.481	5.592	−0.778	−4.518	
B11		0.728	0.227	2.637	−0.589	−3.422	
B8		0.561	−0.266	−3.093	−0.580	−3.371	
B7		0.717	0.554	6.442	−0.537	−3.121	
B4		0.727	0.199	2.309	−0.711	−4.133	
B3		0.763	0.398	4.627	−0.819	−4.759	
B2		0.719	0.243	2.823	−0.810	−4.705	
C12	Exhaustion	0.685	0.298	3.463	−0.774	−4.497	0.660
C8		0.567	−0.045	−0.528	−1.093	−6.353	
C2		0.630	−0.234	−2.715	−1.033	−6.004	
C13	Learning cynicism	0.707	0.800	9.297	−0.044	−0.258	0.847
C10		0.786	1.100	12.783	0.625	3.630	
C9		0.700	0.462	5.371	−0.560	−3.253	
C6		0.730	1.014	11.782	0.591	3.434	
C3		0.722	0.877	10.185	0.399	2.319	
C16	Reduced efficacy	0.787	0.029	0.341	−0.290	−1.684	0.854
C15		0.849	0.130	1.512	−0.316	−1.838	
C14		0.830	0.322	3.736	−0.210	−1.220	
C11		0.565	0.310	3.607	−0.161	−0.937	
C4		0.584	0.306	3.551	−0.215	−1.248	
C1		0.625	0.718	8.343	0.108	0.625	
Multivariate					269.098	71.281	

c.r., corresponding critical ratio; TL, tolerance; WS, withdrawal symptoms; CU, compulsive use; IHP, interpersonal and health-related problems; TMP, time management problems.

As shown in Tables 3, 4, the composite reliability (CR) value were higher than 0.6 (48) and Cronbach’s Alpha (*α*) were higher than 0.7 (49), indicating that the variable had adequate reliability. The convergent validity of the construct is adequate when the Average Variance Extracted (AVE) is higher than 0.5 (48). However, we can accept 0.4 because Fornell and Larcker (48, 50) expressed that if AVE is smaller than 0.5 but CR is higher than 0.6, the convergent validity of the construct is still acceptable. The square roots of the AVE values were higher than the inter-factor correlation coefficients, indicating that the discriminant validity of the factors was verified. Admittedly, latent variables of exhaustion did not have sufficient discriminant validity and reliability (Cronbach’s *α* = 0.660). However, considering the overall integrity of the academic burnout variable, we did not remove the dimensional variable of exhaustion.

**Table 4:** Composite reliability and discriminant validity.

Factor	1	2	3	4	5	6
1.Internet addiction	**0.72**					
2.Social loneliness	0.22	**0.46**				
3.Emotional loneliness	0.43	0.44	**0.47**			
4.Exhaustion	0.46	0.45	0.53	**0.40**		
5.Learning cynicism	0.49	0.50	0.38	0.65	**0.53**	
6.Reduced efficacy	0.44	0.25	0.45	0.40	0.31	**0.51**
CR	0.93	0.87	0.90	0.66	0.85	0.86
Square root of AVE	0.85	0.68	0.69	0.63	0.73	0.72

Values (bold) in diagonal are average variance extracted (AVE), and the off-diagonal values are inter-factor correlations. CR, composite reliability.

The results of the assessment of normality (Table 3) showed that all measurement variables conformed to a normal distribution when the absolute value of skew and kurtosis were ≤ the corresponding critical ratio, in accordance with the requirements of the maximum likelihood method. Hence, we computed the SEM adopting maximum likelihood estimation.

The model fit of *χ*^2^/df = 3.197 ≤ 5, RMSEA = 0.044 ≤ 0.08, estimated after CFA (Table 5), showed that the scales had acceptable structural validity. After verifying and correcting the model, the paths were tested.

**Table 5:** Model fitting step and fitting index.

Step	Model	*χ*^2^/df	RMR	IFI	TLI	CFI	RMSEA
After CFA	Model 0	3.197	0.041	0.911	0.904	0.911	0.052
Hypothesis model	Model 1	3.460	0.062	0.900	0.892	0.900	0.055
Remove path H2c(*t* > 1.96)	Model 2	3.458	0.063	0.900	0.892	0.900	0.055
Add path H4 (MI = 86.417)	Model 3	3.303	0.049	0.907	0.899	0.906	0.053
Remove path H2e (*t* > 1.96)	Model 4	3.302	0.050	0.906	0.899	0.906	0.053
Add path H5(MI = 37.407)	Model 5	3.189	0.044	0.911	0.904	0.911	0.052
Remove path H2a (*t* > 1.96)	Model 6	3.187	0.044	0.911	0.904	0.911	0.052

χ^2^/df, chi-square/degree of freedom ratio; RMR, root mean square residual; IFI, incremental fit index; TLI, the Tucker-Lewis Index; CFI, confirmatory factor index; RMSEA, root mean squared error of approximation.

### 3.5. SEM testing

Based on the theoretical hypothesis and the mediation model above (Figure 2), we fit a structural equation model (SEM) with six latent variables. Based on two model modification indices (51), Modification Index (MI) and the *t*-value, we gradually removed paths that were not statistically significant or added ones that could optimize the model while being theoretically relevant. Specifically, The MI is the minimum chi-square value that can be reduced when adding a specific path (51). The *t*-value is used for model restrictions; the path can be removed if the *t*-value is less than 1.96 (i.e., the parameter estimate is insignificant) (51). However, this approach needs to consider the theoretical basis, whether to remove or add paths (51).

**Figure 3:**
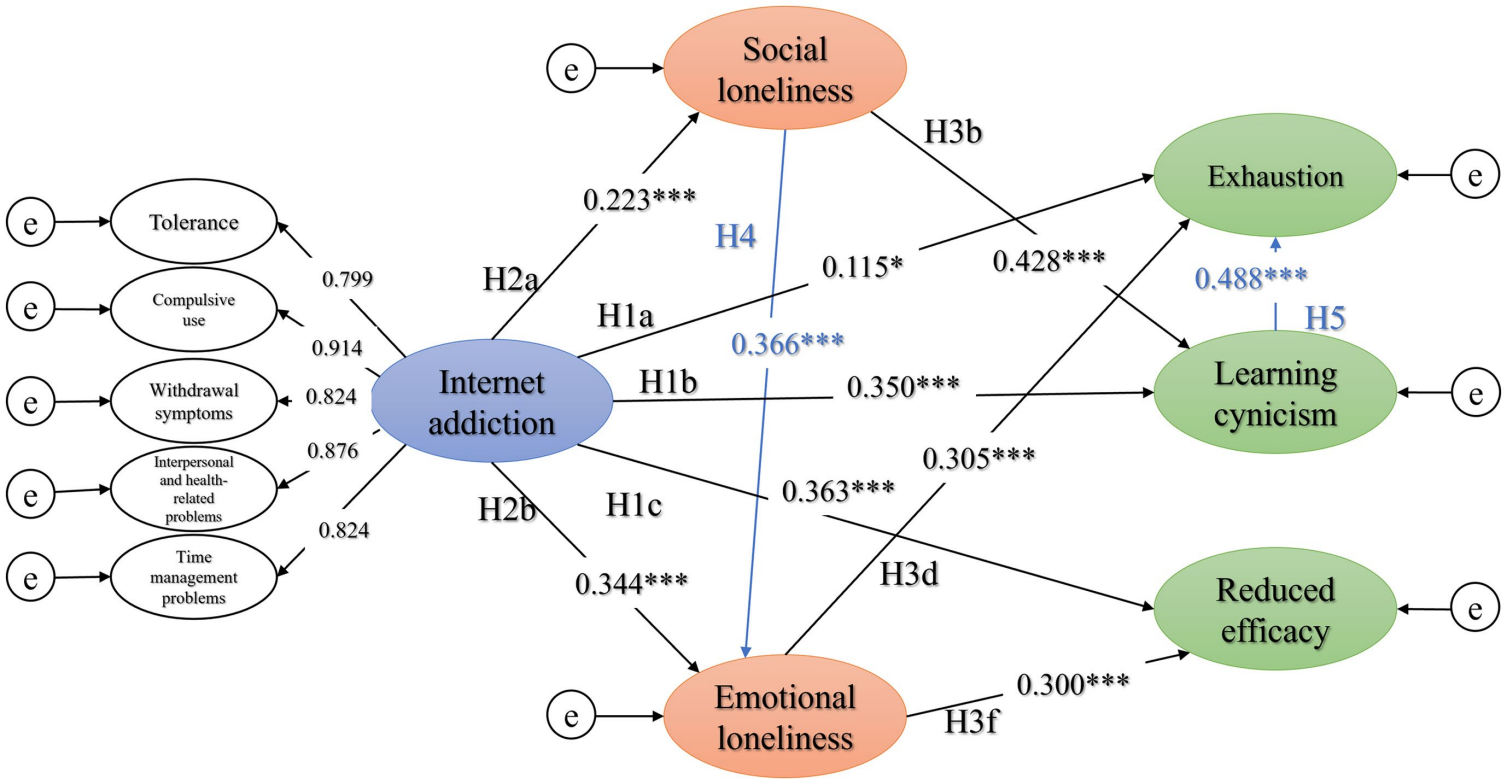
The final structural equation model (model 6). Path coefficients are presented in standardized units, *n* = 810; ***p < 0.001 and *p < 0.05; the variables e represent the structure residuals.

Herein, as shown in Table 5 and Figure 3, we removed three paths that were not statistically significant, namely H2c, H2e, and H2a. Social loneliness could predict emotional loneliness (H4, *β* = 0.366, *p* < 0.001) and learning cynicism may promote exhaustion among Chinese college students (H5, *β* = 0.488, *p* < 0.001). Model 6 (Figure 3) was the last model with *χ*^2^/df = 3.187 and RMSEA = 0.052 (Table 5), indicating that it had a good fit for the observable data.

**Figure 4:**
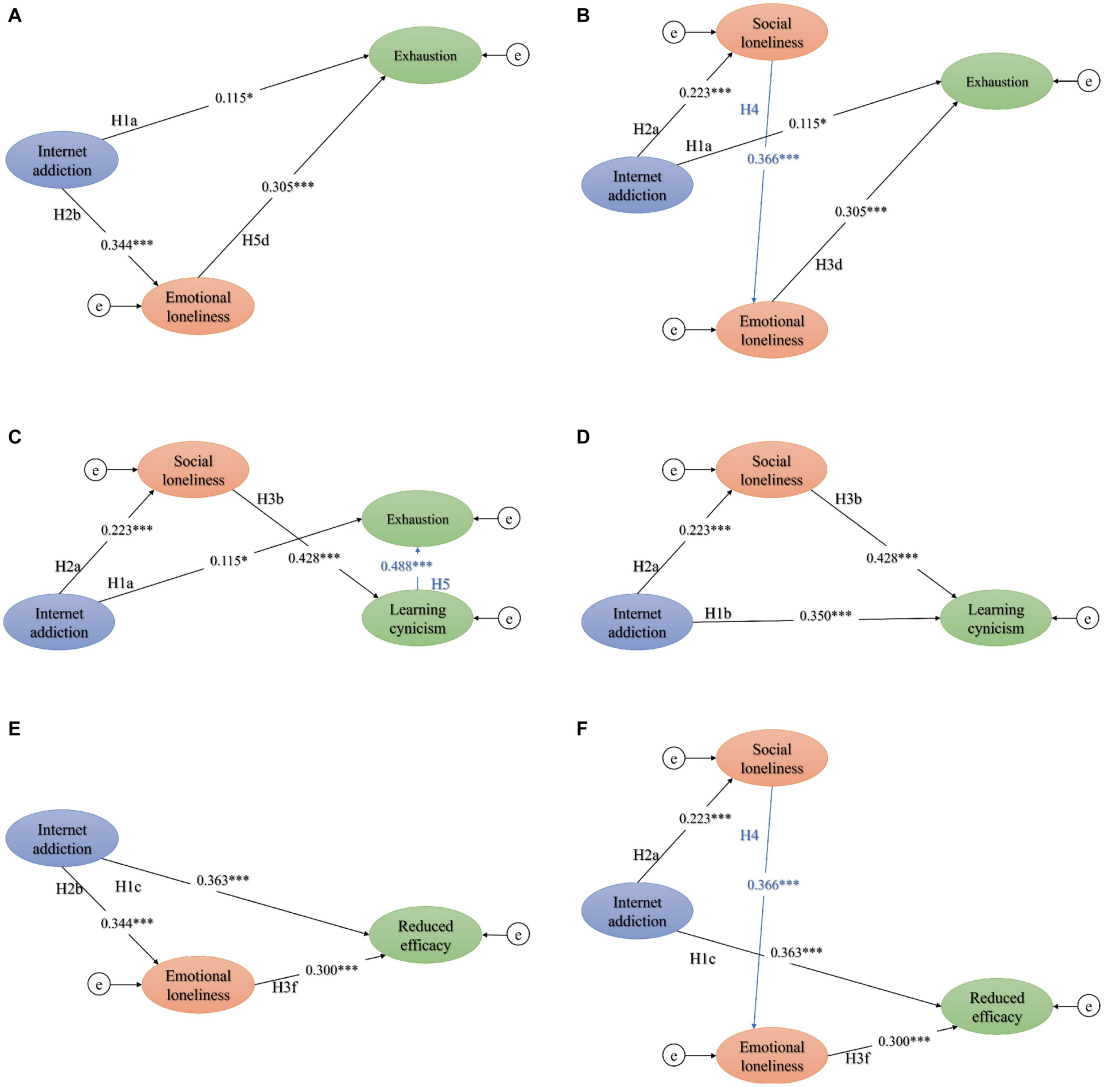
The sub-models of mediating effects in the final model. **(A)** Internet addiction could lead to exhaustion through emotional loneliness; **(B)** Internet addiction could contribute to exhaustion through the chain mediator of “social loneliness via emotional loneliness”; **(C)** Internet addiction could contribute to exhaustion through the chain mediator of “social loneliness to learning cynicism”; **(D)** Internet addiction may indirectly lead to learning cynicism through social loneliness; **(E)** Internet addiction may cause reduced efficacy through emotional loneliness; **(F)** Internet addiction have the effect of mediating the chain of “social loneliness via emotional loneliness,” leading to reduced efficacy. Path coefficients are presented in standardized units, *n*=810; ***p< 0.001, *p< 0.05; The variables 705 e represent the structure residuals.

Overall, our results (Figure 3 and Table 6) were consistent with our hypothesis (Figure 1): (1) Internet addiction directly affect the varying dimensions of academic burnout. The higher the level of Internet addiction, the higher the level of exhaustion (H1a, *β* = 0.115, *p* < 0.05), learning cynicism (H1b, *β* = 0.350, *p* < 0.001), and reduced efficacy (H1c, *β* = 0.363, *p* < 0.001) among students; (2) the final model had six sub-models of mediating effects (Figures 3, 4). For one thing, Internet addiction could lead to exhaustion through emotional loneliness (Figure 4A); in addition to emotional loneliness as a mediator, the chain mediator of “social loneliness via emotional loneliness” (Figure 4B) and the chain mediator of “social loneliness to learning cynicism” (Figure 4C) could also contribute to exhaustion. Second, Internet addiction may indirectly lead to learning cynicism through social loneliness (Figure 4D). Finally, Internet addiction may cause reduced efficacy through emotional loneliness (Figure 4E); likewise, it may have the effect of mediating the chain of “social loneliness via emotional loneliness,” leading to reduced efficacy (Figure 4F). Standardized effects between variables have been presented in Table 6.

**Table 6:** Standardized effects between variables.

Path		Standardized effects (*β*)	Standardized total indirect effects (*β* [bootstrap 95% CI])	Standardized total effects (*β* [bootstrap 95% CI])
Direct	Internet-exhaustion	0.115	–	0.463 [0.355,0.563]
Indirect	Internet-emotional loneliness-exhaustion	0.105	0.347 [0.273,0.424]	
	Internet-social loneliness-emotional loneliness-exhaustion	0.026		
	Internet-social loneliness-learning cynicism-exhaustion	0.048		
	Others	0.168		
Direct	Internet-learning cynicism	0.350	–	0.466 [0.362,0.524]
Indirect	Internet-social loneliness-learning cynicism	0.095	0.095 [0.056,0.137]	
Direct	Internet-learning cynicism	0.363	–	0.491 [0.424,0.553]
Indirect	Internet-emotional loneliness-reduced efficacy	0.103	0.128 [0.086,0.172]	
	Internet-social loneliness-emotional loneliness-reduced efficacy	0.025		

## 4. Discussion

The COVID-19 pandemic has brought considerable changes to our lives. For adolescents in China, the prolonged lockdown and the demands of online learning have led to an increased dependence on the Internet (34, 35). Reports have shown that the pooled prevalence of Internet addiction in medical students was 30.1% as of 2018 (95% confidence interval (CI) 28.5–31.8%) (52). This is much higher than the estimated 19.9% in our survey, which may have been due to the survey instrument. Subgroup analysis shows the pooled prevalence of Internet addiction diagnosed by Chen’s Internet Addiction Scale (CIAS) (5.2, 95% CI: 3.4–8.0%) (52), which is lower than the results of the current survey. The pooled prevalence rate of Internet addiction in healthcare professionals was 9.7% (95% CI: 5.8–13.6%) (53). Internet addiction was associated with healthcare workers’ more significant mental symptom burden and fatigue (53). Results of a study in Southeast Asia showed that the pooled prevalence of Internet addiction was 20% (95% CI: 14.5–27.0%) (54). As such, the prevalence of Internet addiction probably relates to the occupation and survey instrument associated with the study population. Evidence from China has indicated that COVID-19-related isolation and stress could have exacerbated the crisis of Internet addiction (55). Time spent on recreational Internet use increased significantly during the pandemic, and almost half of the participants reported increased severity of Internet addiction (55).

Internet addiction is sometimes associated with academic problems, such as academic stress (56) and performance decrements (57). One variable that adversely affects students’ academic life is burnout (58). Academic burnout includes emotional exhaustion, cynicism, and reduced academic efficiency (59). Several surveys have demonstrated that Internet addiction is positively linked to academic burnout among students (8, 60), which is consistent with our findings (*r* = 0.534, *p* < 0.001). Consequently, we should focus on the prevalence of Internet addiction and academic burnout; however, its related internal mechanisms are rarely reported.

The high prevalence of psychological problems has attracted public attention; loneliness is common among college students (33). During the college years, students face multiple social role changes (61), accompanied by academic, emotional, and work stress, which may bring some risks, such as being alone in a new environment (e.g., a new city) and a lack of familiarity, thereby leading to loneliness. *Loneliness* is synonymous with a situation wherein an individual experiences a subjective lacking in social relationships, either quantitatively or qualitatively (21). According to Weiss (19), there are two types of loneliness: the lack of close and intimate relationships leading to emotional loneliness, and the lack of social networks leading to social loneliness. Our results revealed that 32.8% of college students experienced high loneliness. Loneliness is prevalent among college students, and the finding of predictors of loneliness and its impact on academics provides a theoretical basis for psychological interventions for college students’ academic problems.

Studies have shown that loneliness is closely related to Internet addiction (22–24) and academic burnout (62). This is coherent with the outcomes of our analysis in this survey. Loneliness was positively associated with Internet addiction (*r* = 0.382, *p* < 0.001) and academic burnout (*r* = 0.578, *p* < 0.001), which indicated moderate and strong correlations, respectively (43). To this end, we constructed the mediating model of the three (loneliness, Internet addiction, and academic burnout) and validated the structural equations for different dimensional variables in each of them.

The mediation analysis results verified the previous hypothesis – that loneliness plays a partially mediating role (31.07%) between Internet addiction and academic burnout. Based on this validated model, we further constructed structural equation models to analyze the relationships of different dimensional variables in anticipation of further details on the causal interpretation. Ten direct paths of the model hypothesis were significant in the final model. The model analysis results are discussed below according to the standardized path coefficients of each component.

Two paths were added in the model, namely H4 and H5. Pathway analysis suggests that the two dimensions of loneliness are distinguished yet interconnected. In general, emotional loneliness is characterized by the absence of an attachment figure (as well as feelings of isolation), and social loneliness refers to the lack of a social network that allows one to develop a sense of belonging, of company, and of being part of a community (20). Social loneliness is likely to foster emotional loneliness. The absence of an attachment figure could stem from the lack of a person’s social network. Academic burnout is classified into three dimensions (40): exhaustion refers to the student’s energy exhaustion or emotional exhaustion (40); learning cynicism was described as a gradual loss of enthusiasm for learning-related activities and a negative attitude toward academics (40); the reduced efficacy or inefficacy is when individuals do not experience a sense of accomplishment or efficacy in their studies (40). Our results indicate that having a negative attitude toward academics could trigger emotional exhaustion among college students.

Internet addiction is a direct cause of academic burnout. Prior studies have focused on their correlations (8, 60), but rarely delved into causal associations. The survey revealed reciprocal cross-lagged paths between excessive Internet use and school burnout among adolescents: school burnout predicted further excessive Internet use and excessive Internet use predicted further school burnout (63). This is highly intriguing, considering that Internet addiction and academic burnout intensify during a possible interaction, suggesting that interventions in the process of developing Internet addiction or academic burnout or two-way interventions are possible paths in college students’ education. Our analysis showed that Internet addiction had a significant direct effect on all three dimensions of learning burnout. A longitudinal survey exhibited that the importance of social media website Facebook enhanced the prediction power of changes in the academic burnout total score, exhaustion, and personal inefficacy (64). Compared to the other dimensions, emotional exhaustion was the least significantly directly affected by Internet addiction (H1a, *β* = 0.115, *p* < 0.05), but was significantly influenced by the mediating effect of loneliness (*β* = 0.347, bootstrap 95% CI [0.273, 0.424]), predominantly emotional loneliness. On the other two dimensions of academic burnout, the direct effect of Internet addiction was prominent, accompanied by the partially mediating effect of loneliness. Specifically, Internet addiction could contribute to cynicism through social loneliness rather than emotional loneliness. The reduced efficacy or inefficacy of academic burnout caused by Internet addiction is primarily mediated by emotional isolation. The aforemediated pathways suggested that psychological interventions, especially the psychological regulation of the students’ loneliness, was essential regarding the association between Internet addiction and academic burnout. Internet addiction is the predictor of the three dimensions of academic burnout. Different types of loneliness mediate different types of academic burnout, and the regulation of emotional loneliness should be a high-priority intervention in psychological education for college students.

## 5. Conclusion and limitations

To conclude, Internet addiction could be responsible for academic burnout among college students, in which loneliness plays a partially mediating role. In mediated ways, different types of loneliness contribute to academic burnout. Psychological intervention for loneliness, especially emotional loneliness prevention and control, are critical aspects of Internet addiction accompanied with academic burnout.

This study has limitations. First, the survey was predominantly conducted with first-year medical students since many other senior students went to internships (as presented in Table 1). The characteristics of the respondents may somewhat influence the status of loneliness, Internet addiction, and academic burnout. Still, our study included students with all traits as much as possible, and the above factors would not lead to significant biases in the association between the three. Second, the inventory of academic burnout was aimed at adolescents instead of college students. The scale for academic burnout in the college student population needs further development. Third, this study examined the psychological mechanism of Internet addiction to academic burnout. However, as mentioned earlier (63), the causal relationship between Internet addiction and academic burnout may be a two-way street and, thereby, needs further exploration. Lastly, the study’s findings should be validated in a more representative population in the future. Specifically, the present study was conducted with college students primarily engaged in medical courses, which may somewhat limit the generalization of the study’s findings.

## Data availability statement

The original contributions presented in the study are included in the article/supplementary material, further inquiries can be directed to the corresponding author.

## Ethics statement

The studies involving humans were approved by the ethics review board of Gannan Medical University. The studies were conducted in accordance with the local legislation and institutional requirements. The participants provided their written informed consent to participate in this study.

## Author contributions

JG and YL conceived this study and designed the questionnaire. JG performed data analysis, interpreted the results, and wrote the paper. PW, XH, LF, HL, FL, QX, and XW collected the relevant data. All authors contributed to the article and approved the submitted version.

## Funding

This work was supported by Gannan Medical University (no. Jgkt-2022-35; BKSRW03).

## Conflict of interest

The authors declare that the research was conducted in the absence of any commercial or financial relationships that could be construed as a potential conflict of interest.

## Publisher’s note

All claims expressed in this article are solely those of the authors and do not necessarily represent those of their affiliated organizations, or those of the publisher, the editors and the reviewers. Any product that may be evaluated in this article, or claim that may be made by its manufacturer, is not guaranteed or endorsed by the publisher.
